# Endoplasmic reticulum stress impairs cholesterol efflux and synthesis in hepatic cells[S]

**DOI:** 10.1194/jlr.M043299

**Published:** 2014-01

**Authors:** Clemens Röhrl, Karin Eigner, Katharina Winter, Melanie Korbelius, Sascha Obrowsky, Dagmar Kratky, Werner J. Kovacs, Herbert Stangl

**Affiliations:** * Institute of Medical Chemistry, Center for Pathobiochemistry and Genetics, Medical University of Vienna, Vienna, Austria; † Institute of Molecular Biology and Biochemistry, Center of Molecular Medicine, Medical University of Graz, Graz, Austria; § Institute of Molecular Health Sciences, Department of Biology, Swiss Federal Institute of Technology (ETH) Zurich, Zurich, Switzerland

**Keywords:** ATP-binding cassette transporter A1, apolipoprotein A-I, high density lipoprotein, 3-hydroxy-3-methylglutaryl- coenzyme A reductase, HepG2

## Abstract

Metabolic disorders such as type 2 diabetes cause hepatic endoplasmic reticulum (ER) stress, which affects neutral lipid metabolism. However, the role of ER stress in cholesterol metabolism is incompletely understood. Here, we show that induction of acute ER stress in human hepatic HepG2 cells reduced ABCA1 expression and caused ABCA1 redistribution to tubular perinuclear compartments. Consequently, cholesterol efflux to apoA-I, a key step in nascent HDL formation, was diminished by 80%. Besides ABCA1, endogenous apoA-I expression was reduced upon ER stress induction, which contributed to reduced cholesterol efflux. Liver X receptor, a key regulator of ABCA1 in peripheral cells, was not involved in this process. Despite reduced cholesterol efflux, cellular cholesterol levels remained unchanged during ER stress. This was due to impaired de novo cholesterol synthesis by reduction of HMG-CoA reductase activity by 70%, although sterol response element-binding protein-2 activity was induced. In mice, ER stress induction led to a marked reduction of hepatic ABCA1 expression. However, HDL cholesterol levels were unaltered, presumably because of scavenger receptor class B, type I downregulation under ER stress. Taken together, our data suggest that ER stress in metabolic disorders reduces HDL biogenesis due to impaired hepatic ABCA1 function.

HDL removes excess cholesterol from macrophage foam cells in atherosclerotic plaques and is therefore considered to protect from cardiovascular diseases (1). ApoA-I, the main apolipoprotein of HDL, is secreted by liver and intestine and acquires phospholipids and cholesterol upon interaction with ABCA1 to form nascent, discoid HDL (2–5). The importance of ABCA1 in this process is well established: In mice lacking hepatic ABCA1, reduced HDL levels and increased macrophage foam cell formation are observed (6). In Tangier disease, lack of functional ABCA1 causes severe HDL deficiency (7). Moreover, patients with loss of function mutations in ABCA1 display an increased atherosclerotic burden (8). Adipose tissue ABCA1 contributes to systemic HDL biogenesis (9). In contrast, ABCG1 and scavenger receptor class B, type I (SR-BI) are not found to be necessary for nascent HDL formation in murine primary hepatocytes (10).

Perturbations of endoplasmic reticulum (ER) homeostasis, referred to as “ER stress”, contribute to metabolic dysregulation (11, 12). ER stress is sensed by the unfolded protein response (UPR), a collection of conserved signaling pathways that lead to the adaption of the ER (13–15). In eukaryotic cells, three ER resident proteins are known to sense ER stress: activating transcription factor 6 (ATF6), protein kinase RNA-like ER kinase (PERK), and inositol requiring protein 1 (IRE1) (13). Under normal conditions, glucose-regulated protein 78 (GRP78) binds to the luminal domain of IRE1, ATF6, and PERK to repress the UPR. After ER stress, GRP78 dissociates and causes activation of UPR signaling (15).

A role for ER stress in lipid metabolism is implied by the fact that the activation processes for ATF6 and sterol response element-binding proteins (SREBPs) are similar (16, 17). Indeed, ER stress activates SREBPs. For instance, ER stress induction by homocysteine or thapsigargin activated SREBP in hepatic cells and endothelial cells (18–20). ER stress is also suggested to play a role in steatosis in animal models and in human patients (21–24). Elevated saturated fatty acids lead to hepatic ER stress (25), and a recent study showed that lack of adipose triglyceride lipase protects from ER stress in the liver (26). This effect was attributed to alterations in hepatic fatty acid composition. Although the role of cholesterol-mediated ER stress in macrophages during atherosclerosis is under extensive investigation (27–30), knowledge about the role of ER stress in hepatic cholesterol metabolism is limited. Recent work suggests that accumulation of hepatic ER cholesterol exerts ER stress and might influence the development of insulin resistance (31).

Hepatic ER stress is associated with metabolic disorders, in particular type 2 diabetes mellitus, insulin resistance, and obesity (32). These disorders are characterized by dyslipidemia and low HDL levels. Reduced apoA-I transcription and increased HDL clearance might partially account for the reduction in HDL levels (33–35). In this paper we addressed the question of whether deregulations of cholesterol metabolism in hepatic cells by ER stress negatively influence HDL metabolism. A negative impact of ER stress on HDL metabolism might help to explain dyslipidemia in metabolic disorder. Our results show that ER stress deregulates cholesterol metabolism in human hepatic cells by impairing ABCA1-mediated cholesterol efflux and cholesterol synthesis via reduction of HMG-CoA reductase activity.

## EXPERIMENTAL PROCEDURES

### Cell culture

Cells were cultivated under standard conditions. HepG2 cells (HB-8065; ATCC, Manassas, VA) were grown in MEM supplemented with 10% FBS, 1% penicillin/streptomycin, and 1% nonessential amino acids (all from PAA, Pasching, Austria). HuH7 (ATCC: JCRB-0403) cells were maintained in DMEM containing 10% FBS and 1% penicillin/streptomycin. If not otherwise indicated, experiments were performed in the respective growth media containing lipoprotein-deficient serum (LPDS) instead of FBS to exclude interference with extracellular lipid sources. LPDS was prepared from FBS as described (36). Thapsigargin, tunicamycin, TO-901317, and lovastatin were from Sigma-Aldrich (St. Louis, MO).

### Gene expression analysis

RNA was isolated using the RNeasy Plus Micro Kit (Qiagen, Düsseldorf, Germany), and cDNA was synthesized from 2 µg RNA. qRT-PCR was performed using the following TaqMan probes (Life Technologies, Carlsbad, CA): CCAAT-enhancer-binding protein homologous protein (CHOP) (Hs01090850_m1), ATF4 (Hs00909569_g1), GRP78 (Hs00607129_gH), ER-localized DnaJ-domain containing protein 4 (ERdJ4) (Hs01052402_m1), ABCA1 (Hs00194045_m1), apoA-I (Hs00985000_g1), ABCG5 (Hs00223686_m1), inducible degrader of the LDL receptor (IDOL) (Hs00203131_m1), LDLR (Hs00181192_m1), 3-hydroxy-3-methylglutaryl-CoA reductase (Hs00168352_m1), and 18s (Hs99999901_s1).

### Western blot analysis

Proteins were isolated and equal amounts were separated by PAGE and transferred to nitrocellulose membranes (Sigma). After blocking, membranes were incubated with primary antibodies (anti-ABCA1, ab18180; anti-β-actin, ab8229; anti-apoA-I, ab7613 [all from Abcam, Cambridge, UK]; and anti-SRBI, NB400-104 [Novus]) at 4°C over night. Membranes were then incubated with the appropriate HRP-coupled secondary antibodies followed by detection using the Super Signal chemiluminescence system (Thermo Scientific, Rockford, IL) and a Chemilmager 4440 (Biozym, Oldendorf, Germany).

### Immunofluorescence microscopy

Cells were fixed with 4% formaldehyde in PBS for 30 min, permeabilized with ice-cold methanol for 20 s, and blocked with 2.5% BSA in PBS for 30 min. Samples were incubated with primary antibodies (see Western blot analysis) diluted 1:250 in 1% BSA and 1% horse serum in PBS for 60 min and then washed with 1% BSA and 0.1% TWEEN-20 in PBS. Cells were incubated with the appropriate secondary antibodies coupled to Alexa^488^ (Life Technologies) diluted 1:250 in 1% BSA and 1% horse serum in PBS for 60 min, followed by washing with 1% BSA and 0.2% TWEEN-20 in PBS. Finally, samples were counterstained with DAPI, mounted with Fluoprep (Biomerieux, Paris, France), and visualized with an Axiovert microscope (Zeiss, Jena, Germany).

### Cholesterol efflux assays

Cells were seeded in 24-well plates on day 0 and trace labeled with 1 µCi/ml ^3^H-cholesterol (Perkin Elmer, Waltham, MA) in media containing 10% FBS on day 1. On day 2, cells were thoroughly washed with PBS and equilibrated in media for 2 h. Cells were then incubated with or without thapsigargin, 10 µg/ml apoA-I, and 5 µM T0901317 in media containing 2 mg/ml fatty acid-free BSA for 24 h. Alternatively, cholesterol efflux measurements were performed in media containing 10% LPDS with or without 0.1 µM thapsigargin or 5 µM T0901317 for 24 h. Supernatants were collected, centrifuged to remove cell debris, and analyzed by β-counting (Tri-Carb 2800 TR, Perkin Elmer). Cells were washed with PBS and lysed with 0.1 M NaOH, and cell lysates were analyzed by β-counting. Cholesterol efflux was calculated by dividing supernatant counts by total counts (i.e., supernatant plus cell lysate counts) and expressed in percent.

### Promoter activity assay

ABCA1 reporter constructs were provided by Dr. Gerd Schmitz (University Hospital Regensburg, Germany). The ABCA1 core promoter (−175/+224) had been cloned into the pGL3-basic vector encoding firefly luciferase (37). The luciferase reporter vector containing three sterol regulatory elements (38) was provided by Dr. Michiyo Amemiya-Kudo (Okinaka Memorial Institute for Medical Research, Japan).

A pGL4.73 vector coding for renilla luciferase (Promega, Madison, WI) under control of a constitutive TK promoter served as a transfection control. Cells were seeded in 24-well plates on day 0 and cotransfected on day 1 with 1 µg/well reporter vector and 100 ng/well control vector using Nanofectamin (PAA) according to the manufacturer's instructions. Cells were treated with thapsigargin 4 h later, and promoter activity was measured after another 24 h using the Dual Luciferase Assay system (Promega). ABCA1 promoter activity was normalized to control vector activity.

### GC

GC was used to directly quantify the cellular content of free cholesterol and cholesteryl esters within a single run (39). Cells were detached using trypsin, and lipids were isolated from cell pellets by standard Folch extraction. An aliquot of the pellet was used for cell protein determination by the Bradford assay. The analyses were carried out on a GC-2010 gas chromatograph (Shimadzu, Kyoto, Japan) equipped with a programmed temperature vaporizer injector and a fused silica capillary column (DB-5; 12 m length; 0.25 mm inner diameter). Tridecanoyl glycerol and cholesteryl myristate (Sigma-Aldrich) were used as standards for free and esterified cholesterol, respectively. The chromatograms were quantified using GC Solutions 2.3 (Shimadzu), and results were normalized to cell protein.

### Filipin staining

Cells were fixed with 4% formaldehyde in PBS at 4°C for 30 min and stained with 50 µg/ml Filipin III (Sigma-Aldrich) diluted in PBS containing 10% LPDS at RT for 30 min. Cells were washed, mounted, and visualized with an Axiovert microscope (Zeiss).

### Lipid synthesis from acetate

Cells were seeded in 6 cm dishes on day 0 and treated with thapsigargin in media containing LPDS on day 2. After 0, 4, 8, or 24 h, cells were trace-labeled with 1 µCi/ml ^14^C-acetate (Perkin Elmer). One hour later, lipids were extracted from cell monolayers using hexane/isopropanol (3/2). Directly before lipid extraction, ^3^H-oleic acid was added as recovery marker to the cell monolayers to allow for compensation of sample loss during lipid extraction and TLC. Lipid extracts were concentrated and separated by TLC on silica gel sheets (Polygram SIL G; Marcherey-Nagel, Düren, Germany) using heptane/diethyl ether/acetic acid (90/30/1) as solvent. Spots were detected by iodine vapor (Rf values: 0.11 for FC and 0.69 for CE), excised, and analyzed by liquid scintillation counting. Values were normalized to the recovery marker.

### HMG-CoA reductase activity

Cells were seeded in 6 cm dishes on day 0 and incubated with media containing LPDS on day 1. After 24 h, cells were treated with thapsigargin in media containing LPDS for another 16 h. Afterward, cells were lysed, and HMG-CoA reductase activity was monitored by measuring synthesis of ^14^C-mevalonate from ^14^C-HMG-CoA as described (40) with the following modifications: Cells were lysed with KEND buffer plus 0.1% Triton X-100 and 1% protease inhibitor cocktail (Sigma) and homogenized using a syringe and a 26G needle. Mevalonate synthesis was measured over 1 h. HMG-CoA reductase activity was calculated by subtracting blank values as determined by reactions performed without cell lysates. Values were normalized to cell protein as determined by the Bradford assay.

### Animal experiments

Animal experiments were approved by the Austrian Ministry of Science and Research, Division of Genetic Engineering and Animal Experiments, Vienna, Austria (BMWF-66.010/0159-II/3b/2012). Male, 25 week old C57BL/6 mice fed a standard chow diet (Ssniff, Soest, Germany) were intraperitoneally injected with 1 mg tunicamycin per kg body weight (injected as a solution of 0.1 mg tunicamycin per ml PBS). Control mice were injected with PBS only. The injection was repeated after 24 h, mice were euthanized after another 24 h, and livers were isolated. Gene expression was analyzed by qRT-PCR and Western blot analysis as described above. Plasma was collected after 24 h. Plasma total cholesterol was determined using an enzymatic test kit (Greiner Bio-One, Kremsmünster, Austria). To determine the cholesterol content of individual lipoprotein fractions, pooled plasma of four animals was separated by gel filtration using an ÄKTA fast-protein liquid chromatography system (GE Healthcare, Fairfield, CT) equipped with a superpose-6 column at a flow rate of 0.1 ml/min. Fractions of 0.3 ml were collected and analyzed for total cholesterol using a fluorometric test kit (Cayman, Ann Arbor, MI).

### Statistics

Statistical analysis was performed using GraphPad Prism v4.00. A two-sided *t*-test or ANOVA was used to compare two or more groups, respectively.

## RESULTS

### Induction of ER stress impairs ABCA1 expression

To investigate a putative link between hepatic ER stress and HDL levels, we examined the expression of ABCA1, which is crucial in HDL biogenesis, in human HepG2 hepatoma cells after induction of acute ER stress. For induction of ER stress, we applied thapsigargin, which disturbs intracellular calcium homeostasis, or tunicamycin, which impairs protein glycosylation (41, 42).

**Fig. 1.**
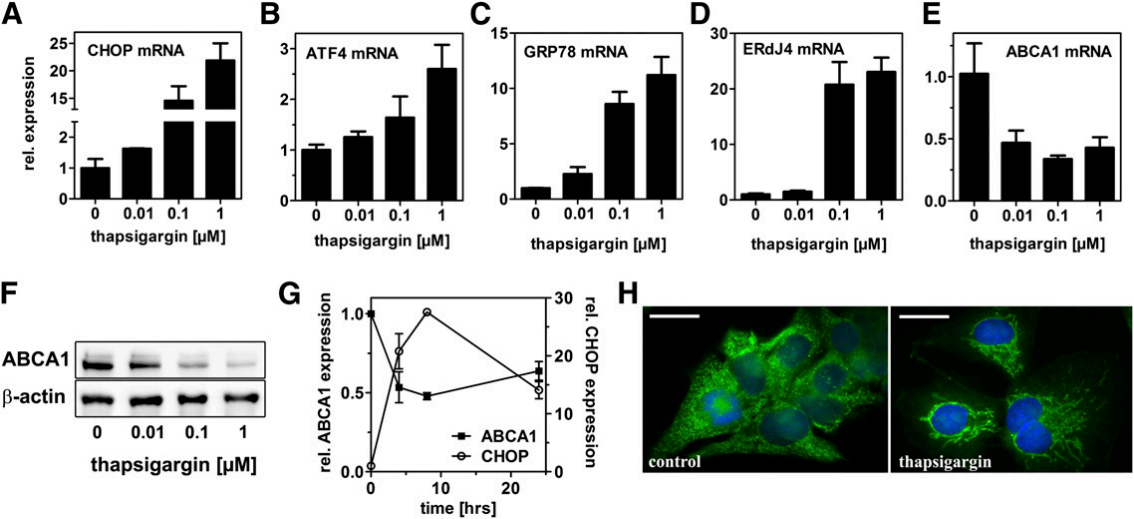
Induction of ER stress by altering calcium homeostasis impairs ABCA1 expression and redistributes ABCA1. HepG2 cells were incubated with thapsigargin in media containing 10% LPDS for 24 h. mRNA expression was determined by qRT-PCR and normalized to 18s expression. The ER stress markers CHOP, ATF4, GRP78, and ERdJ4 are induced dose dependently, whereas ABCA1 mRNA was reduced (A–E). Western blot analysis showed dose-dependent reduction of ABCA1 protein expression by thapsigargin treatment (F). ABCA1 and CHOP mRNA expression is altered inversely over time after thapsigargin (0.1 µM) treatment (G). Thapsigargin treatment (0.1 µM) alters ABCA1 localization to tubular cellular compartments as visualized by immunofluorescence microscopy (h). Green: ABCA1; blue: DAPI; bar = 5 µm. qRT-PCR: mean ± SD (n = 3). For Western blot and immunofluorescence analyses, representative images from three independent experiments are shown.

HepG2 cells were incubated with thapsigargin for 24 h in media containing LPDS instead of FBS to avoid interference with extracellular cholesterol donors and acceptors. As expected, thapsigargin treatment induced expression of several UPR genes dose dependently (**Fig. 1A–D**) : increased CHOP and ATF-4 expression revealed activation of the PERK pathway. Increased expression of the ER chaperone GRP78 and ERdJ4, an IRE1 downstream target, also indicates ER stress induction by thapsigargin. Concomitantly, ABCA1 mRNA was reduced by thapsigargin, yielding a 70% reduction at a concentration of 0.1 µmol/l (Fig. 1E). Decreased ABCA1 mRNA was accompanied by a dose-dependent reduction of ABCA1 protein expression (Fig. 1F). After ABCA1 mRNA expression over time, an inverse expression pattern compared with CHOP expression was observed (Fig. 1G). ABCA1 mRNA expression decreased over time, with the greatest reduction seen after 8 h. When CHOP expression declined again after 8 h, indicating adaption to ER stress, ABCA1 expression levels increased. Because ABCA1 localization is important for its proper function, we further investigated its localization by immunofluorescence. Indeed, thapsigargin treatment induced redistribution of ABCA1 to tubular perinuclear compartments (Fig. 1H).

**Fig. 2.**
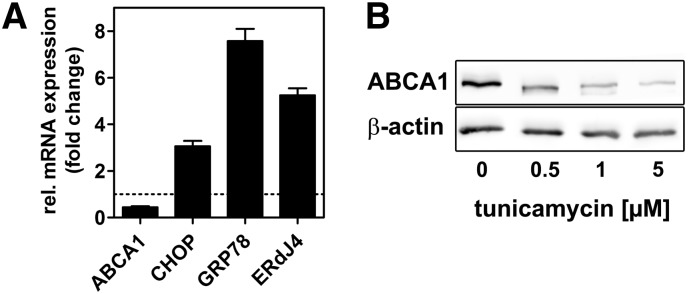
Induction of ER stress by inhibiting protein glycosylation reduces ABCA1 expression. HepG2 cells were incubated with tunicamycin in media containing 10% LPDS for 24 h. mRNA expression was determined by qRT-PCR and normalized to 18s expression. Tunicamycin treatment (1 µM) reduces ABCA1 mRNA expression, whereas the ER stress markers CHOP, GRP78, and ERdJ4 are increased (A). Tunicamycin treatment reduces ABCA1 protein expression dose dependently (B). qRT-PCR: mean ± SD (n = 3). For Western blot analysis, a representative image from three independent experiments is shown.

As an independent approach to induce ER stress, HepG2 cells were treated with tunicamycin. Tunicamycin treatment similarly reduced ABCA1 mRNA and protein expression, whereas expression of CHOP, GRP78, and ERdJ4 was increased (**Fig. 2**). Finally, induction of ER stress by thapsigargin in human hepatic HuH7 cells and immortalized human hepatocytes and in murine hepatic Hepa1-6 cells reduced ABCA1 expression similar to our observations in HepG2 cells (supplementary Fig. I). Altogether, these data suggest a general role for ER stress in ABCA1 regulation in hepatic cells, irrespective of the cell line or the mechanism of ER stress induction.

### Induction of ER stress impairs cholesterol efflux and apoA-I expression

**Fig. 3.**
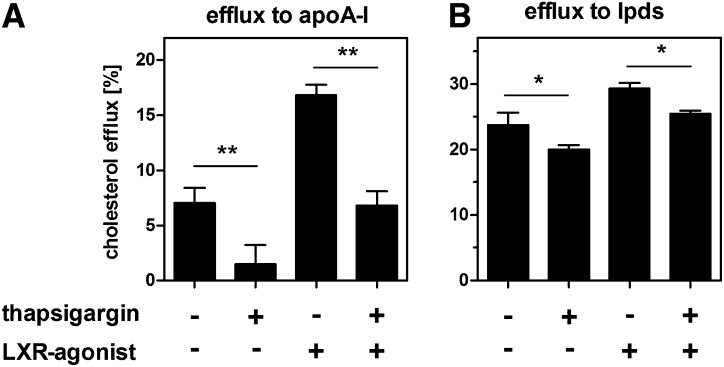
ER stress reduces cholesterol efflux. HepG2 cells were trace-labeled with ^3^H-cholesterol. Cholesterol efflux was measured for 24 h in media containing 2 mg/ml fatty acid-free BSA and 10 µg/ml apoA-I with or without 0.1 µM thapsigargin or 5 µM of the LXR agonist T0901317 (A). Alternatively, cholesterol efflux measurements were performed in media containing 10% LPDS with or without thapsigargin or T0901317 (B). Under both conditions, thapsigargin treatment reduces cholesterol efflux, even in the presence of the synthetic LXR agonist. Data show means ± SD from three experiments.

**Fig. 4.**
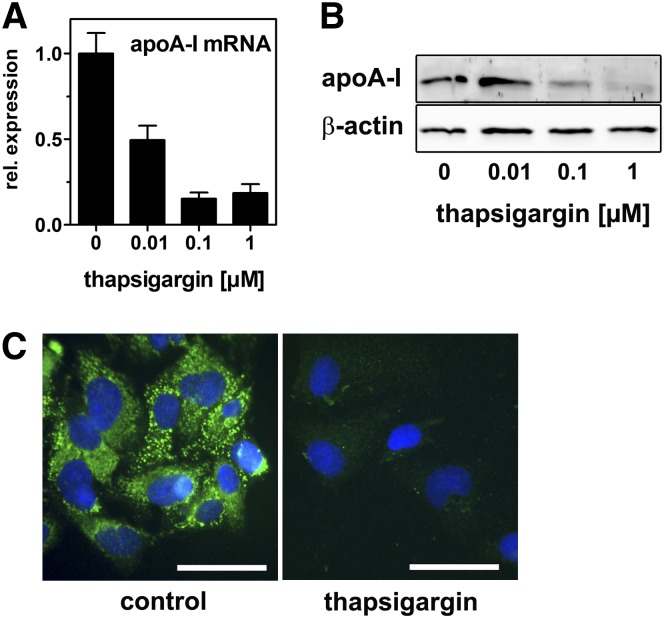
ER stress impairs apoA-I expression. HepG2 cells were incubated with increasing concentrations of thapsigargin (A, B) or 0.1 µM thapsigargin (C) in media containing 10% LPDS for 24 h. ApoA-I mRNA was determined by qRT-PCR and normalized to 18s expression (A). ApoA-I protein expression was analyzed by Western blotting (B) and immunofluorescence analyses (C). Green: ABCA1; blue: DAPI. Bar = 10 µm. apoA-I mRNA and protein are decreased by thapsigargin treatment. qRT-PCR: mean ± SD (n = 3). Western blot and immunofluorescence analysis: representative images from two independent experiments are shown.

To elucidate the functional relevance of impaired ABCA1 expression and localization by ER stress, we analyzed cholesterol efflux to apoA-I. Thapsigargin treatment significantly reduced cholesterol efflux even when cholesterol efflux was induced by a synthetic liver X receptor (LXR)-agonist (**Fig. 3A**). In contrast, cholesterol efflux to HDL was unaltered (data not shown). However, cholesterol efflux was impaired when experiments were performed in media containing LPDS (Fig. 3B). The finding that ER stress modulates cholesterol efflux to LPDS without the addition of exogenous apoA-I may be explained by the presence of residual acceptors for ABCA1 in the LPDS (e.g., free apoA-I or albumin) or by the reduced efflux to endogenously synthesized and secreted apoA-I. Indeed, thapsigargin treatment reduced apoA-I mRNA (**Fig. 4A**). Consistently, Western blot and immunofluorescence analyses revealed decreased endogenous apoA-I protein expression (Fig. 4B, C). Our data indicate that ER stress reduces the expression of ABCA1 and apoA-I to coordinately impair cholesterol efflux.

### ABCA1 is regulated independently of LXR

**Fig. 5.**
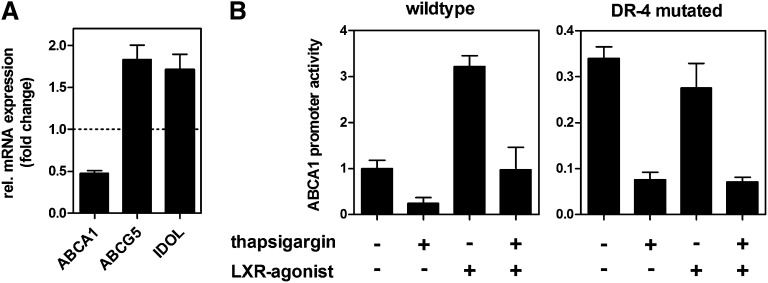
ABCA1 is regulated independently of LXR during ER stress. A: HepG2 cells were treated with 0.1 µM thapsigargin in media containing 10% LPDS for 24 h. Gene expression was determined by qRT-PCR and normalized to 18s expression. mRNA expression of ABCA1 was compared with expression of ABCG5 and IDOL, which are established LXR target genes in hepatic cells. ABCA1 is regulated differently compared with ABCG5 and IDOL. Data show means ± SD from three experiments. B: HepG2 cells were transfected with luciferase reporter vectors containing wild-type or LXR-binding site (DR-4) mutated constructs of the ABCA1 core promoter (−175/+224). After 4 h, cells were treated with 0.1 µM thapsigargin and/or 5 µM of LXR agonist TO901317 in media containing 10% LPDS for another 24 h. Promoter activity was determined using dual luciferase assay. Data show one representative experiment out of three independently performed experiments. Promoter activity was normalized to the activity of the wild-type construct under untreated conditions.

LXR is the major transcriptional regulator of ABCA1 expression in peripheral cells such as macrophages. In contrast, the role of LXR in hepatic ABCA1 expression is unclear. To address if ER stress regulates ABCA1 expression via LXR in HepG2 cells, we compared expression of ABCA1 with ABCG5 and IDOL, which are established LXR target genes in liver cells (43, 44). **Fig. 5A** shows that thapsigargin treatment reduces ABCA1 mRNA expression to 50%, whereas expression of ABCG5 and IDOL increases approximately 1.7-fold. This discrepancy suggests that LXR is activated by ER stress and does not account for decreased ABCA1 expression. To further substantiate LXR-independent ABCA1 regulation, we analyzed ABCA1 promoter activity in wild-type and LXR binding site (DR-4) mutated constructs. ER stress reduced ABCA1 promoter activity to 25% irrespective of the presence of a functional LXR binding site (Fig. 5B). As expected, a synthetic LXR agonist induced ABCA1 promoter activity in the wild-type construct only. Taken together, these data suggest that ER stress regulates ABCA1 expression by a transcriptional mechanism independent of LXR.

### ER stress alters free cholesterol distribution but not cholesterol content

**Fig. 6.**
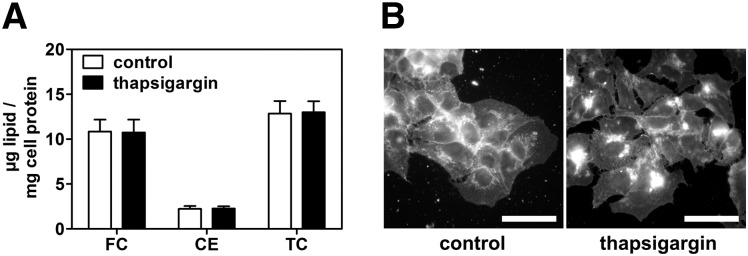
ER stress alters cholesterol distribution but not cholesterol content. HepG2 cells were treated with 0.1 µM thapsigargin in media containing 10% LPDS for 24 h. A: Lipids were extracted, and free and esterified cholesterol were directly quantified by GC. Data show means ± SD from three experiments. CE, esterified cholesterol; FC, free cholesterol; TC, total cholesterol. B: Free cholesterol distribution was visualized by Filipin staining. Cholesterol distribution is altered by ER stress, although no quantitative alterations in FC and CE exist. Bar = 10 µm. Representative images from three independent experiments are shown.

We tested if impaired cholesterol efflux influences the cellular free and esterified cholesterol content. Unexpectedly, quantitative analysis by GC revealed that neither free nor esterified cholesterol content was altered when HepG2 cells were treated with thapsigargin in the presence of LPDS (**Fig. 6a**). However, free cholesterol was redistributed to perinuclear cellular compartments as shown by Filipin staining (Fig. 6B). These results show that ER stress impairs cholesterol efflux without concomitant accumulation of cellular cholesterol. Consequently, we analyzed if ER stress decreases cholesterol synthesis.

### ER stress decreases cholesterol synthesis despite enhanced SREBP-2 activity

**Fig. 7.**
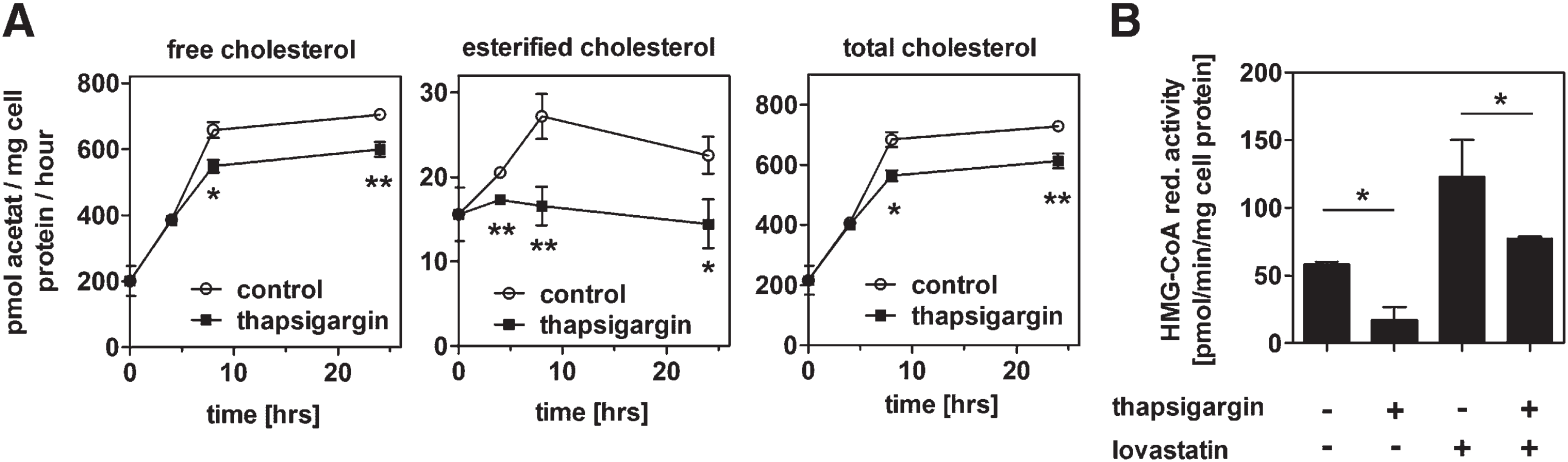
ER stress represses cholesterol synthesis. A: HepG2 cells were treated with 0.1 µM thapsigargin in media containing 10% LPDS for 0, 4, 8, or 24 h. Afterward, incorporation of ^14^C-acetate into free and esterified cholesterol was measured for 1 h. One representative experiment out of three independent experiments is shown. B: HepG2 cells were incubated with media containing 10% LPDS for 24 h. Afterward, cells were treated with 0.1 µM thapsigargin in media containing 10% LPDS for another 16 h. HMG-CoA reductase activity was measured as described in the experimental procedures section. De novo synthesis of free and esterified cholesterol as well as HMG-CoA reductase activity decreases after thapsigargin treatment, even when cells were treated with 10 µM lovastatin to induce HMG-CoA reductase activity. Data show means ± SD from three experiments.

To analyze the effect of ER stress on cholesterol synthesis, we monitored the incorporation of acetate into free and esterified cholesterol. **Fig. 7A** shows that the free cholesterol synthesis rate increases over time in control cells in response to the withdrawal of exogenous cholesterol due to LPDS treatment. This increase was significantly less pronounced in thapsigargin-treated cells (Fig. 7A). In addition, the synthesis rate of esterified cholesterol was lower after thapsigargin treatment. However, formation of esterified cholesterol was quantitatively low and did not significantly contribute to total cholesterol synthesis.

We next measured the activity of HMG-CoA reductase, the rate-limiting enzyme in the cholesterol biosynthetic pathway. HMG-CoA reductase activity was decreased to 30% by ER stress (Fig. 7B). This decrease was also apparent when HMG-CoA reductase activity was increased as a compensatory effect to lovastatin treatment. This downregulation of cholesterol synthesis by ER stress is in line with the finding that the cellular cholesterol content is unaltered despite impaired cholesterol efflux.

**Fig. 8.**
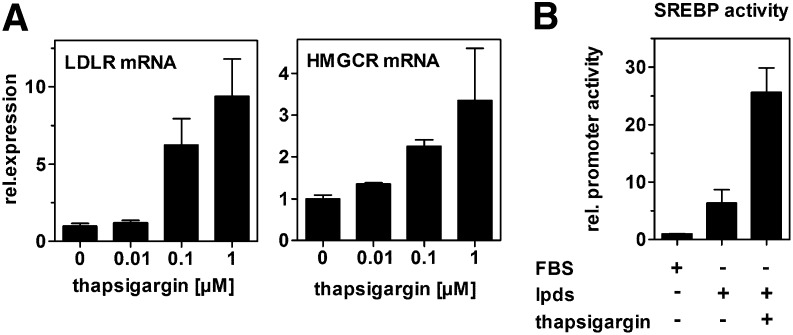
ER stress increases SREBP-2 activity. A: HepG2 cells were incubated with thapsigargin in media containing 10% LPDS for 24 h. mRNA expression of the established SREBP-2 target genes LDL-receptor and HMGCR was determined by qRT-PCR and normalized to 18s expression. Data show means ± SD from three experiments. B: HepG2 cells were transfected with luciferase reporter vectors containing the SREBP response element. After 4 h, cells were incubated in media containing 10% FBS or 10% LPDS or 10% LPDS plus 0.1 µM thapsigargin for another 24 h. Promoter activity was determined using dual luciferase assay. Withdrawal of exogenous cholesterol by LPDS treatment increases SREBP-2 activity, which is further increased in response to thapsigargin treatment. Data show one representative experiment out of three independently performed experiments.

Current literature suggests that ER stress induces SREBP activity. Consistently, ER stress induction by thapsigargin treatment resulted in a dose-dependent upregulation of the established SREBP-2 target genes LDL-receptor and HMG-CoA reductase (**Fig. 8A**). Moreover, we measured SREBP activity using reporter vectors containing copies of the SREBP binding site. SREBP activity increased as expected when cells were switched from FBS to LPDS (Fig. 8B). This effect was even more pronounced when thapsigargin was added. Therefore, ER stress increases SREBP-2 activity as shown by target gene expression analysis and activity assays. Altogether our data indicate that ER stress impairs cholesterol synthesis despite SREBP-2 activation, suggesting posttranscriptional mechanisms for HMG-CoA reductase inhibition.

### ER stress impairs ABCA1 expression in the liver of mice

**Fig. 9.**
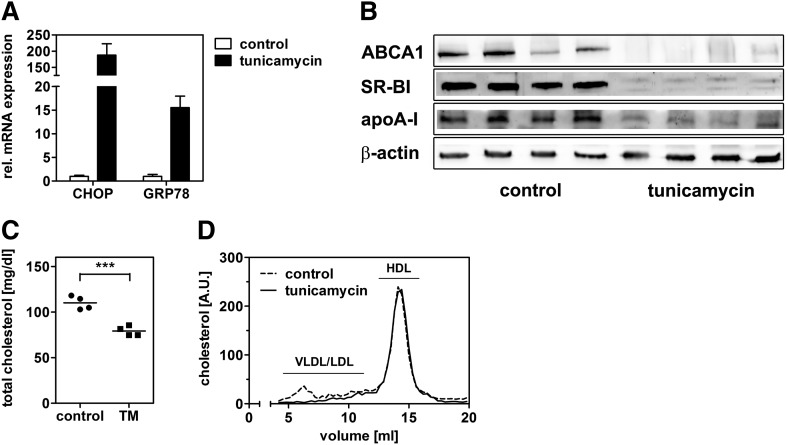
ER stress reduces ABCA1 expression in the murine liver. C57BL/6 mice were intraperitoneally injected with 1 mg/kg bodyweight tunicamycin or solvent (n = 4). Mice were euthanized after 48 h. Liver RNA and protein were isolated after 48 h and analyzed by qRT-PCR (A) and Western blot analysis (B). Expression of ABCA1, apoA-I, and SR-BI are decreased upon ER stress induction. Plasma total cholesterol was quantified (C), and relative cholesterol distribution was analyzed in pooled plasma after separation by fast-protein liquid chromatography (D). The reduction of plasma cholesterol can be attributed to decreased VLDL/LDL cholesterol levels.

We aimed to validate our key findings obtained in hepatic cells lines in mice. We injected tunicamycin, which is widely used to exert ER stress in vivo, into mice. Tunicamycin treatment induced expression of CHOP and GRP-78 in the liver, indicating hepatic ER stress (**Fig. 9A**). In addition, ABCA1 and apoA-I protein expression levels were strongly reduced (Fig. 9B), which confirms the in vitro data. Moreover, SR-BI expression was markedly decreased. Plasma total cholesterol was lower in mice treated with tunicamycin compared with untreated controls (79 vs. 110 mg/dl). However, this decrease was due to reduced cholesterol content in the VLDL/LDL fraction, whereas HDL cholesterol was unaltered (Fig. 9C, D).

Taken together, our in vitro and in vivo data demonstrate that hepatic ER stress impairs ABCA1 expression.

## DISCUSSION

In this study we provide evidence for novel links between hepatic ER stress and cholesterol metabolism. We show that acute ER stress impairs cholesterol efflux and synthesis in hepatic cells. Induction of ER stress in HepG2 cells was accomplished by two independent approaches using thapsigargin, which depletes luminal calcium as well as tunicamycin, which inhibits protein glycosylation. Both approaches decreased ABCA1 expression in a comparable manner (Fig. 1 and 2). In addition, apoA-I expression was reduced (Fig. 4). The reduction of ABCA1 and apoA-I resulted in reduced cholesterol efflux (Fig. 3). These findings were validated in mice where ER stress was induced by tunicamycin injection. Hepatic ER stress in vivo was associated with reduced ABCA1 and apoA-I expression (Fig. 9). Thus, we hypothesize that hepatic ER stress negatively influences HDL biogenesis. However, ER stress induction in mice did not influence plasma HDL levels. Presumably, reduced HDL biogenesis through impaired ABCA1 expression is accompanied by reduced HDL cholesterol clearance due to reduced SR-BI expression (Fig. 9).

ABCA1 regulation in hepatic cells is incompletely understood. In peripheral cells, sterol loading induces activation of LXR, which upregulates ABCA1 expression and enhances cholesterol efflux. In contrast, sterols repress ABCA1 transcription in rat hepatoma cells (45), and cholesterol feeding does not induce ABCA1 expression in parenchymal liver cells (46). Furthermore, ABCA1 expression is not reduced in LXRα knockout mice (47); together, these findings suggest a minor regulatory role of cholesterol and LXR on hepatic ABCA1. In line with these findings, our data show transcriptional, LXR-independent regulation of ABCA1 during ER stress (Fig. 5) because an ABCA1 promoter construct lacking an LXR-binding site was efficiently downregulated. Thus, the mechanisms of ABCA1 transcriptional downregulation during ER stress remain to be established. In addition to a transcriptional mechanism, ABCA1 expression might be regulated by a posttranscriptional mechanism by ER stress. Indeed, 1 µM thapsigargin reduced ABCA1 mRNA expression by 60%, whereas ABCA1 protein expression was reduced by 90% (Fig. 1). Moreover, ER stress induction in mice considerably reduced ABCA1 protein expression (Fig. 9) without causing significant changes of ABCA1 mRNA (data not shown). Advanced glycated albumin, which induces ER stress, reduced ABCA1 protein expression without altered ABCA1 transcription in macrophages (48).

An important regulatory factor of hepatic ABCA1 regulation is microRNA (miR)-33, which links SREBP-activation to reduced ABCA1 expression (49). Accordingly, we analyzed miR-33 expression after ER stress induction. miR-33 expression was unaltered by thapsigargin treatment in HepG2 cells (data not shown). Moreover, our data clearly show a reduction in the activity of promoter constructs derived from the 5′ untranslated region of ABCA1 (Fig. 5), whereas miR-33 targets the 3′ untranslated region of ABCA1 (49). This finding further suggests that miR-33 does not play a major role in the repression of ABCA1 under ER stress.

Acute ER stress is physiologically relevant because it is found, for instance, after infusion of physiological doses of carbohydrates or lipids (50). However, chronic ER stress, where ER homeostasis cannot be fully restored, is of significant relevance to the development of metabolic disorders (12). Two studies potentially link chronic ER stress with reduced ABCA1 expression: The group of Arnold von Eckardstein found reduced ABCA1 expression in the liver and in peritoneal macrophages of diabetic mice (51). In subsequent in vitro studies, they identified unsaturated fatty acids and acetoacetate as negative regulators of ABCA1 expression in HepG2 and RAW264.7 macrophages. On the basis of our data, we hypothesize that ER stress is involved in the observed downregulation of ABCA1 in HepG2 cells and diabetic mice. Furthermore, ABCA1 expression is reduced in leukocytes and cultured skin fibroblasts from patients with diabetes (52). In addition, ABCA1 protein concentration and function were associated with HDL-C levels. Again, ER stress-mediated downregulation of ABCA1 might explain these findings.

Our data on reduced apoA-I expression upon ER stress are partly in line with recent findings of Naem and coworkers (53), who found reduced apoA-I protein expression and secretion after treating HepG2 cells with tunicamycin and thapsigargin. However, the authors report no alterations in apoA-I mRNA levels, suggesting posttranscriptional apoA-I degradation. In contrast, our data clearly indicate reduced apoA-I mRNA upon ER stress (Figs. 4 and 9).

In addition to reduced cholesterol efflux, we observed reduced cholesterol synthesis after ER stress induction, although SREBP-2 activity was induced (Figs. 7 and 8). This leads to unaltered cellular cholesterol levels (Fig. 6) in spite of impaired cholesterol export, at least in the absence of exogenous cholesterol donors. Although our study is the first to directly show impaired cholesterol synthesis by ER stress, the link between ER stress and SREBP-2 activation had been the subject of previous studies. In peroxisome-deficient PEX2^−/−^ mice, SREBP-2 is activated independently of cholesterol, an effect that was attributed to increased ER stress in these animals (54). Furthermore, SREBP-2 activation by ER stress was reported in HepG2, HeLa, and MCF7 cells and in the liver of hyperhomocysteinemic mice (19, 20). These authors reported cellular accumulation of lipids, including cholesterol; however, they did not directly analyze cholesterol synthesis, and increased cholesterol was likely due to increased LDL uptake. In our study, we performed experiments in the absence of exogenous cholesterol and could directly observe decreased cholesterol synthesis by ER stress. In contrast to the aforementioned studies and our data, ER stress induction by glucose deprivation or ATF6 overexpression repressed SREBP-2 activity and lipid accumulation in HepG2 cells (55). The reason for this discrepancy is unknown.

Although SREBP-2 activity and HMG-CoA reductase mRNA were increased, HMG-CoA reductase activity was reduced, which led to decreased cholesterol synthesis (Figs. 7 and 8). Volpe and Goldberg (56) observed that tunicamycin reduces HMG-CoA reductase activity. However, the authors reported that impaired glycoprotein synthesis reduced HMG-CoA reductase activity and did not link their findings to ER stress. HMG-CoA reductase expression is considerably regulated by posttranscriptional mechanisms. In particular, HMG-CoA reductase degradation by the endoplasmic reticulum-associated degradation (ERAD) pathway is an important process in the regulation of its expression (57, 58). Because ER stress induction is linked with increased ERAD activity via IRE1 and PERK (59), we speculate that posttranscriptional degradation of HMG-CoA reductase by ERAD is increased under acute ER stress conditions and leads to repressed cholesterol synthesis. We found that HMG-CoA reductase activity was decreased by 70% under ER stress, whereas cholesterol synthesis was decreased by 16% (Fig. 7). This discrepancy might be rationalized by upregulation of cholesterol biosynthetic enzymes other than HMG-CoA reductase, many of which are SREBP-2 target genes and might be induced by ER stress (60).

Although ER stress affects cholesterol efflux and synthesis, our data suggest that the regulatory mechanisms leading to impaired cholesterol efflux and reduced cholesterol synthesis are not tightly linked to each other. Under physiological conditions, reduced cholesterol efflux is expected to lower endogenous synthesis because transiently accumulating cholesterol represses SREBP-2 activity. Under ER stress, however, we found increased SREBP-2 activity, suggesting that impaired cholesterol efflux does not repress cholesterol synthesis via the canonical SREBP-2 pathway. Likewise, reduced cholesterol synthesis does not mediate ABCA1 downregulation under ER stress. First, we found that ABCA1 is not regulated by LXR (Fig. 5), and therefore cholesterol is not a main determinant of ABCA1 expression in hepatic cells. Second, we did not observe reduced ABCA1 expression when we blocked cholesterol synthesis by lovastatin treatment (data not shown). Thus, it is unlikely that ABCA1 is downregulated due to impaired cholesterol synthesis.

ER stress altered subcellular distribution of ABCA1 and free cholesterol (Fig. 1 and 6). ER stress alters vesicular trafficking from the ER to the Golgi as well as Golgi architecture and anterograde transport (61, 62). Thus, proper ABCA1 glycosylation and transport to the plasma membrane might be altered—besides its reduced expression—upon ER stress. This might lead to impaired ABCA1 function. In addition, we found that the nonvesicular cholesterol transport proteins StarD4 and StarD5 are upregulated after thapsigargin treatment (data not shown), which is in agreement with previous studies (63–65). Thus, alterations in Golgi compartments and increased expression of nonvesicular cholesterol transport proteins might explain the perinuclear accumulation of cholesterol. Whether this redistribution of cholesterol is functional in terms of availability for efflux or for increased SREBP-2 activity remains to be investigated.

Taken together, our data show severe alterations in cellular cholesterol metabolism by ER stress. These findings might be physiologically relevant to the development of dyslipidemia in metabolic disorders, which are associated with hepatic ER stress.

## Supplementary Material

Supplemental Data
