# Supplementary material for: Endoplasmic reticulum stress impairs cholesterol efflux and synthesis in hepatic cells
Source: J Lipid Res. 2014 Jan;55(1):94–103. doi: 10.1194/jlr.M043299 (PMC3927476; doi:10.1194/jlr.M043299)
Supplement: Supplemental Data [file supp_55_1_94__index.html]

Endoplasmic reticulum stress impairs cholesterol efflux and synthesis in hepatic cells — Endoplasmic reticulum stress impairs cholesterol efflux and synthesis in hepatic cells — Supplemental Data 

# Endoplasmic reticulum stress impairs cholesterol efflux and synthesis in hepatic cells

## Supplemental Data

**Files in this Data Supplement:**

- Supplemental Figure S1 - ER stress reduces ABCA1 expression in several hepatic cell lines.
