# Supplementary material for: Endoplasmic reticulum stress impairs cholesterol efflux and synthesis in hepatic cells
Source: J Lipid Res. 2014 Jan;55(1):94–103. doi: 10.1194/jlr.M043299 (PMC3927476; doi:10.1194/jlr.M043299)
Supplement: Supplemental Data [file supp_M043299_jlr.M043299-1.docx]

SUPPLEMENTAL INFORMATION

Endoplasmic reticulum stress impairs cholesterol efflux and synthesis in hepatic cells*

**Clemens Röhrl, Karin Eigner, Katharina Winter, Melanie Korbelius, Sascha Obrowsky, Dagmar Kratky, Werner J. Kovacs and Herbert Stangl**

**Figure S1.** Induction of ER stress in hepatic cell lines reduces ABCA1 expression. HuH7 human hepatoma cells, immortalized human hepatocytes (IHH; [1]) and Hepa1-6 mouse hepatoma cells were incubated with thapsigargin in media containing 10% lpds for 24 hrs. mRNA expression was determined by qRT-PCR and normalized to 18s expression. In all three cell lines, thapsigargin treatment reduces ABCA1 mRNA expression, whereas the expression of the ER stress marker CHOP is increased. Graphs show mean +/- SD; n=3.





1. Schippers IJ, Moshage H, Roelofsen H, Muller M, Heymans HS, et al. (1997). Immortalized human hepatocytes as a tool for the study of hepatocytic (de-)differentiation. Cell Biol Toxicol 13: 375-386.
